# Practical Hints for Operations on the Ear

**Published:** 1909-04-03

**Authors:** 


### Anæsthetics.

### PRACTICAL HINTS FOR OPERATIONS ON THE EAR.

For short aural operations, such as incision of the membrane, nitrous oxide is in the majority of cases a good anæsthetic on account of absence of after-effects and freedom from danger. It should, of course, not be used when contra-indicated by such conditions as respiratory or circulatory disorder. Generally it proves fairly convenient for the surgeon, although when complete stillness of the patient is necessary it is not always entirely satisfactory. There is apt to be trouble from movement of the head, which interferes with the concentration of light on the very limited area of operation and with the delicacy of the surgeon's touch. The operator should, before administration is begun, place the head in the desired position, and it should be firmly held there by an assistant, not by the anæsthetist, who cannot properly attend to this and to his own duties as well, especially when a prolongation of the anæsthesia is needed.

For operations in the mastoid region, unless the patient is feeble, it is better to give chloroform, or a mixture containing it, than ether, provided, of course, that the administrator is fully competent. The bone and overlying tissues are often very vascular, and bleed so freely if ether be given that the surgeon's difficulties are considerably increased. A large piece of gauze should be arranged with a view to preventing the operator's fingers or instruments accidentally touching the face-piece. The anæsthetist should be at the side opposite the surgeon, with the assistant standing between them.

During the latter part of the operation the anæsthetist should be on the look-out for twitching of the facial muscles, and should at once tell the surgeon if it occurs. This must be distinguished from grimaces due to insufficient depth of anæsthesia.

Usually only a light anæsthesia is needed whilst bone is being removed, but it is well to deepen it on approach of the final stage of the operation, when more sensitive structures (skin and subcutaneous tissues) are being cut or stitched, otherwise sudden movement may occur. If morphine have been given beforehand, or if the patient be inclined to coma, extreme caution is required. Only enough anæsthetic should be given to prevent movement, and the respiration should be very carefully watched.

